# Sublethal Pyriproxyfen Exposure Alters *Anopheles arabiensis* Fitness and Pyrethroid Susceptibility Without Trans-Generational Carry-Over

**DOI:** 10.3390/insects17020166

**Published:** 2026-02-02

**Authors:** Simoni Twaha Mnzava, Augustino Thabiti Mmbaga, Anitha Mutashobya, Letus Laurian Muyaga, Mwema Felix Mwema, Halfan Ngowo, Dickson Wilson Lwetoijera

**Affiliations:** 1 Environmental Health and Ecological Sciences Department, Ifakara Health Institute, Ifakara P.O. Box 53, Tanzania; ammbaga@ihi.or.tz (A.T.M.); amutashobya@ihi.or.tz (A.M.); letus@ihi.or.tz (L.L.M.); hngowo@ihi.or.tz (H.N.); 2 School of Life Sciences and Bio-Engineering, The Nelson Mandela African Institution of Sciences and Technology, Arusha P.O. Box 447, Tanzania; 3 School of Materials, Energy, Water and Environmental Sciences, The Nelson Mandela African Institution of Science and Technology, Arusha P.O. Box 447, Tanzania; mwema.felix@nm-aist.ac.tz

**Keywords:** pyriproxyfen, *Anopheles arabiensis*, autodissemination, pyrethroids, insecticides, sublethal dose, semi-field settings

## Abstract

Background: Pyriproxyfen (PPF), a juvenile hormone analog, is a promising chemical for autodissemination strategies, where mosquitoes aid in transferring insecticides to their breeding sites. This study evaluated the effects of sublethal PPF doses on *Anopheles arabiensis* fitness (fecundity, fertility, and body size) and pyrethroids susceptibility across three generations. Methods: Laboratory-reared, pyrethroid-resistant mosquito larvae were exposed once to sublethal PPF concentrations of (0.0003, 0.0006, 0.0008, and 0.001 mg a.i./L), which caused emergence inhibition rates of 5%, 10%, 15%, and 20%, respectively, alongside a control group. Emerged adults were tested for susceptibility to 0.75% permethrin, 3.75% permethrin, and 0.05% deltamethrin using WHO tube bioassays. Knockdown was recorded at 1 h, mortality at 24 h post-exposure, while fecundity, fertility, and body size were measured across three generations. Results: First-generation *Anopheles arabiensis* emerging from PPF-treated larvae showed reduced susceptibility to 0.75% permethrin and 0.05% deltamethrin compared to controls. Mortality from permethrin dropped from 22.7% in the controls to 11% and then 3% at 0.0003 and 0.001 mg a.i./L. For deltamethrin, mortality fell from 62.7% to 36% and then 23.3% at the same concentrations. Knockdown at 60 min was also significantly reduced, with permethrin KDT_60_ dropping from 41.3% in controls to 9.3% and 3.3%, and deltamethrin KDT_60_ from 79.7% to 66.7% and 65%. No significant differences were observed in subsequent generations (*p* > 0.05). PPF exposure also induced notable fitness costs in the first generation: mean wing length decreased from 3.07 mm in controls to 2.88–2.66 mm (6–13% reduction), mean egg production dropped from 30.1 to 13.9–18.8 eggs per female (37–54% reduction), and egg hatching rate declined from 87% to 79–82% (6–9% reduction). Conclusions: These findings suggest that sublethal PPF doses can temporarily enhance insecticide resistance without leading to heritable resistance and negatively impact key mosquito fitness traits. PPF may thus be a valuable addition to integrated vector management strategies.

## Simple Summary

Malaria is spread by mosquitoes and controlling their population is an important way to reduce the disease. Scientists are testing new tools to help fight mosquitoes, especially as they become resistant to commonly used insecticides. One such tool is a chemical called pyriproxyfen, which works by interfering with mosquito development and reproduction. In this study, we exposed mosquito larvae to very small amounts of pyriproxyfen and observed the effects over three generations. We found that mosquitoes exposed to this chemical became temporarily tolerant to insecticides, but this effect is not transmissible to the next generation. We also saw that the exposed mosquitoes laid fewer eggs, had lower egg hatching rates, and were smaller in size. These results show that pyriproxyfen may be useful in mosquito control programs because it weakens mosquitoes without causing trans-generational carry-over. This approach could help improve the effectiveness of existing malaria control strategies and protect public health.

## 1. Background

Malaria remains a major problem, with an estimated 263 million cases globally in 2023, an increase of 11 million from the previous year. This rise underscores the threat malaria poses, especially in regions with limited resources [1,2]. According to the World Malaria Report 2024, sub-Saharan Africa continues to bear a disproportionate share of the global malaria burden, accounting for approximately 95% of malaria cases and deaths worldwide [2]. Controlling mosquito-borne diseases remains vital for public health and the well-being of vulnerable communities in sub-Saharan African countries. Alarming statistics reveal that Tanzania is among the four countries in sub-Saharan Africa accounting for over half of the worldwide malaria-related deaths [2,3].

Long-lasting insecticide-treated nets (LLINs) and indoor residual spraying (IRS) have long been the cornerstone of malaria control in Africa [2,4]. However, the effectiveness of these indoor interventions is increasingly threatened by the emergence of insecticide resistance in targeted mosquito populations [2,5]. This growing resistance necessitates the exploration of alternative vector control strategies to sustain malaria control efforts.

To complement LLINs and IRS, many African countries are now adopting larval source management (LSM) as a Supplementary Malaria control strategy [2,6,7,8,9,10]. LSM involves applying chemical or biological products to mosquitoes’ breeding habitats to eliminate immature stages before they develop into adult vectors [2,11]. Unlike LLINs and IRS, which primarily target adult mosquitoes, LSM focuses on preventing the emergence of adult mosquitoes. By targeting breeding sites, LSM complement existing malaria control efforts and contributes towards the elimination goal [2,7,10].

For effective larviciding, WHO has recommended its use in areas where mosquito breeding habitats are few, fixed, and findable [1]. This presents a major challenge in rural and peri-urban areas, where mosquito breeding sites are often numerous, hidden in nature and difficult to access [12]. Additionally, the widespread adoption of LSM is hindered by the high operation costs. As an alternative, the use of Unmanned Aerial Vehicles (UAVs) has improved the identification and treatment of breeding habitats in rural settings, surpassing the limitations of human-based larviciding efforts [12,13]. However, despite its potential, UAV-based larviciding remains costly and requires significant technical expertise, which limits its large-scale adoption [12,14].

Given these challenges, there is a pressing need for complementary cost-effective larviciding approaches. One promising solution is the autodissemination technique using PPF, an approach that baits mosquitoes themselves to transfer insecticides to their preferred breeding sites [15,16]. In this strategy, mosquitoes pick up a lethal dose of PPF from contaminated artificial resting sites and transfer it to their breeding habitats during oviposition, ultimately inhibiting the emergence of mosquitoes therein [15]. Several studies have demonstrated the effectiveness of PPF autodissemination in controlling *Aedes*, *Culex*, and *Anopheles* mosquitoes in laboratory, semi field, and field settings [15,16]. This method exploits mosquito behavior to achieve high coverage of breeding habitats. It requires minimal human effort, uses less insecticide, and incurs low associated labor costs [17,18].

Pyriproxyfen is a juvenile hormone analog and insect growth regulator (IGR) that effectively disrupts the development of mosquitoes [19,20]. It mimics the action of natural juvenile hormones, interfering with mosquito development and preventing larvae from maturing into adults [21]. So far, previous studies have examined PPF and pyrethroids in combination with LLINs and IRS [22,23,24,25,26,27,28]. While there is currently no documented evidence of PPF resistance in malaria vectors, in vitro studies suggest that PPF can be metabolized by P450 enzymes, similar to pyrethroids [22]. A recent evidence by Opiyo et al., indicates that sublethal doses of PPF may amplify pyrethroid resistance in mosquitoes that already exhibit early signs of pyrethroid resistance [23]. However, the assessed effect of PPF exposure on pyrethroid susceptibility was in a single generation, with uncertainty as to whether the observed resistance was temporary (tolerance) or actual resistance. To address this gap, this study assessed the effects of sublethal PPF exposure on *An. arabiensis* fitness parameters and susceptibility to pyrethroid insecticides over multiple generations.

## 2. Methods

### 2.1. Study Area

The study was conducted from March to November 2024 at Kinin’gina village, rural southeastern Tanzania (8.11417° S, 36.67484° E). Experiments were carried out in the semi field system (SFS) using laboratory reared *An. arabiensis*. A detailed description of the SFS at Ifakara Health Institute and its validation for entomological studies has been described elsewhere [29,30].

### 2.2. Mosquito Rearing

Third-instar larvae of pyrethroid-resistant *An. arabiensis* were obtained from the IHI insectary. This colony originated from field collections in Sakamaganga village, Ifakara, southern Tanzania, and has been maintained since 2011. The strain exhibits established resistance mechanisms, including kdr mutations and upregulation of cytochrome P450 enzymes. In June 2024, WHO susceptibility bioassays indicated 24% and 52% mortality when exposed to discriminating doses of permethrin and deltamethrin, respectively, confirming a high level of resistance. During all experiments, larvae were reared on TetraMin^®^ (Blacksburg, VA, USA) fish food twice daily, and adults were maintained on a 10% sucrose solution *ad libitum*. For egg laying, female mosquitoes were starved for 12 h prior to blood feeding, which was conducted by allowing them to feed on the arm of a consenting human volunteer for 30 min on two consecutive days to ensure full engorgement.

### 2.3. Tested Insecticide

PPF powder containing 50% active ingredient (a.i.) with a particle size of 20 to 63 µm, manufactured by Ban Field Bio Inc., Woodinville, WA, USA, was used in all experiments.

### 2.4. Treatments with Pyriproxyfen

To establish test sublethal concentrations, a larval bioassay using twenty-five laboratory-reared third-instar larvae of *An. arabiensis* exposed in 200 mL of either untreated water (control) or PPF concentrations (0.00001, 0.0001, 0.0005, 0.0008, 0.001, 0.0015, 0.002, 0.01, 0.025, 0.05, 0.1, and 0.5 mg/L) were conducted. Each concentration was replicated four times. Larvae were fed with TetraMin^®^ fish food twice daily. Using WHOPES guidelines, the number of live larvae, pupae, and emerged adults were recorded every 24 h until all larvae had either died or emerged [31]. Test PPF concentrations of 0.0003, 0.0006, 0.0008, and 0.001 mg/L, that resulted to 5%, 10%, 15%, and 20% adult emergence inhibition, respectively, were selected for the follow-up experiment. In the main experiment, concentrations of 5% and 20% were used for rearing larvae used in the pyrethroid susceptibility bioassay experiment, while concentrations of 5%, 10%, 15%, and 20% were used for rearing larvae used in the fitness experiment. All test concentrations were prepared by serially diluting stock solution of 0.1 mg AI/L.

### 2.5. Effect of Pyriproxyfen Exposure on Insecticide Susceptibility

To assess the effect of larval exposure to sublethal doses of PPF on adult insecticide susceptibility, 3000 third-instar larvae of pyrethroid-resistant *An. arabiensis* were equally divided into six basins containing 2 L of water. Larvae were exposed to either 0.0003 mg/L or 0.001 mg/L PPF, or to untreated water. Adults emerging from PPF treated and untreated basins in the first generation were pooled and maintained in 35 × 35 × 35 cm net cages. Following blood feeding, the second and third generations larvae were not exposed to PPF. Emerged adults, that were labeled as treatment and control groups, were kept separately and provided with 10% glucose for WHO standard susceptibility assays. For each generation, 150 unfed mosquitoes of 3–5-day-old females from each treatment and control group were exposed to 0.75% permethrin, 0.05% deltamethrin, and 3.75% permethrin (28). Six replicates of 25 mosquitoes per replicate were performed for each test insecticide, with respective controls of non-impregnated papers. To establish the effect of PPF, similar procedures were performed for adult mosquitoes reared in untreated water.

### 2.6. Effect of Pyriproxyfen Exposure on Fitness Parameters

To assess the effects of PPF on mosquito fitness exposed to sublethal doses, 2000 third-instar larvae in 2 L of water were exposed to each PPF concentrations of 0.0003, 0.0006, 0.0008, 0.001 mg/L, and untreated water (control). This setup was replicated four times. Emerged adults from each group in the first generation were kept in net cages and blood-fed to produce second and third generations that were reared without PPF. In each generation, 30 blood-fed *An. arabiensis* females (5–9 days old) from each treatment and control group were individually placed in oviposition cups lined with damp filter paper 72 h post-blood feeding. Mosquitoes were maintained on 10% glucose solution *ad libitum* during oviposition monitoring. Oviposition cups were observed for up to seven days, and once eggs were detected, the filter paper was removed, the eggs were counted under a stereomicroscope and transferred into basins containing 2 L of water. Eggs were monitored daily for three consecutive days to record hatching. Fecundity was determined by the number of eggs laid, while fertility was calculated as the proportion of eggs that hatched.

After oviposition, females were killed by freezing for 10 min, and one wing was removed, mounted on a slide, and measured (from the apical notch to the axillary margin) under a microscope using a micrometer to estimate body size [32].

### 2.7. Statistical Analysis and Presentation

The results were analyzed using open source software, R version 4.5.1 [33].

Experiment 1: Dose–response analysis

To evaluate emergence inhibition, a log dose–response analysis was performed. This analysis estimated the concentrations of PPF required to achieve 5%, 10%, 15%, and 20% emergence inhibition rates.

Experiment 2: Effect of sublethal PPF exposure on susceptibility to pyrethroids

The effect of sublethal PPF exposure on susceptibility to pyrethroids was evaluated using standard WHO bioassays with permethrin (0.75% and 3.75%) and deltamethrin (0.05%) across three mosquito generations. The outcomes assessed were knockdown at 60 min and 24 h mortality, both modeled as binary responses. Binomial Generalized Linear Mixed Models (GLMMs) were fitted using the glmer function in the lme4 package [34], with PPF concentration, generation, and insecticide type as fixed effects, and replicate batches included as random intercepts. Separate models were fitted for each insecticide, and interactions between generation and PPF concentration were tested. Descriptive statistics (mean ± SD) and odds ratios with 95% confidence intervals were reported.

Experiment 3: Effect of sublethal PPF exposure on mosquito fitness

Fitness parameters assessed included fecundity, hatchability, and wing length across three generations. For fecundity, data showed zero inflation and skewness, confirmed by Shapiro–Wilk testing. Poisson GLMs were fitted separately for each generation, as well as an overall model testing the Generation × Concentration interaction. Predictions were generated with ggeffects and visualized in ggplot2.

Hatchability was analyzed using binomial GLMs fitted per generation, as well as an overall model that included the Concentration × Generation interaction. Overdispersion was tested and appropriately accounted for. Model results were expressed as odds ratios with 95% confidence intervals, and predicted proportions hatched (with 95% confidence intervals) were generated using ggeffects and visualized in ggplot2.

Wing length was treated as a continuous trait. Data were first tested for normality using the Shapiro–Wilk test, which showed mild skewness. Accordingly, Gamma GLMs with a log-link were fitted to model the effects of PPF exposure. Both separate and interaction models were fitted to assess PPF effects across generations. Means were predicted with 95% confidence intervals and visualized in ggplot2.

## 3. Results

### 3.1. Initial Tests to Determine the Range of Sublethal PPF Doses

**Figure 1:**
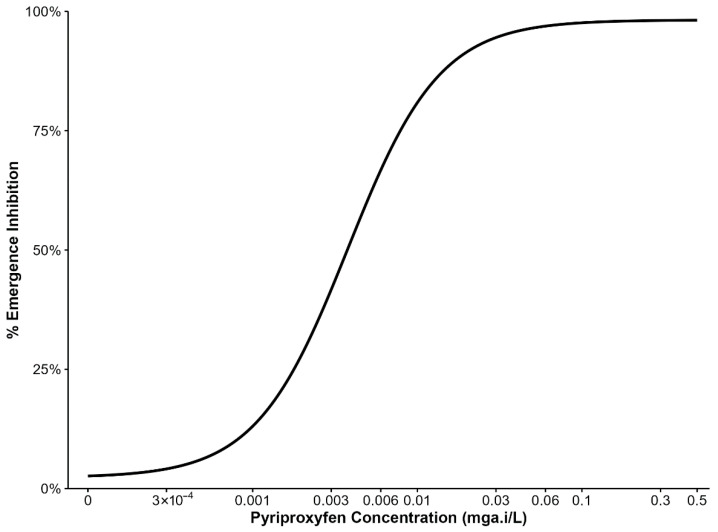
Percentage inhibition of adult mosquito emergence from larvae reared with different concentrations of pyriproxyfen.

Emergence inhibition of third-instar larvae exposed to various PPF doses are summarized in (Figure 1). Data from four replicates of the *An. arabiensis* colony were pooled per dose to estimate the concentrations inhibiting 5%, 10%, 15%, and 20% adult emergence, which were used in subsequent experiments. PPF doses between 3 × 10^−4^ mg a.i/L and 0.003 mg a.i/L caused 5% and nearly 50% emergence inhibition. Therefore, subsequent experiments used doses in the range of 3 × 10^−4^ mg a.i/L to 0.001 mg a.i/L.

### 3.2. Effect of Sublethal Dose of PPF on An. arabiensis Susceptibility to Pyrethroids

In the first generation, adult mosquitoes emerging from larvae exposed to PPF showed significantly reduced mortality to both permethrin and deltamethrin. For permethrin, mortality decreased from 22.7% in the control to 11.0% at 0.0003 mg a.i./L (OR = 0.77; 95% CI: 0.60–0.97; *p* = 0.026) and to 3.0% at 0.001 mg a.i./L (OR = 0.66; 95% CI: 0.52–0.82; *p* < 0.001). For deltamethrin, mortality declined from 62.7% in the control to 36.0% at 0.0003 mg a.i./L (OR = 0.34; 95% CI: 0.24–0.47; *p* < 0.001) and to 23.3% at 0.001 mg a.i./L (OR = 0.18; 95% CI: 0.13–0.26; *p* < 0.001) (Table 1).

In the second generation, no significant differences were observed. Permethrin mortality was 22.7% in the control, compared with 22.0% at 0.0003 mg a.i./L (*p* = 0.813) and 23.0% at 0.001 mg a.i./L (*p* = 0.953). For deltamethrin, mortality was 60.3% in the control and remained similar at 58.3% (0.0003 mg a.i./L, *p* = 0.617) and 60.0% (0.001 mg a.i./L, *p* = 0.933) (Table 1).

In the third generation, mortality also remained comparable to controls. Permethrin mortality was 25.0% in the control, 22.7% at 0.0003 mg a.i./L (*p* = 0.953), and 24.7% at 0.001 mg a.i./L (*p* = 0.694). For deltamethrin, control mortality was 62.0%, with 59.3% at 0.0003 mg a.i./L (*p* = 0.504) and 61.3% at 0.001 mg a.i./L (*p* = 0.867) (Table 1). However, exposure to 3.75% permethrin resulted in 100% knockdown and mortality across all PPF concentrations and controls in all generations.

A similar pattern was observed for knockdown at 60 min (KDT_60_) across generations. In the first generation, PPF exposure significantly reduced knockdown for both insecticides. For permethrin, knockdown decreased from 41.3% in the control to 3.3% at 0.001 mg a.i./L (OR = 0.44, 95% CI: 0.35–0.56, *p* < 0.001) and 9.3% at 0.0003 mg a.i./L (OR = 0.50, 95% CI: 0.39–0.64, *p* < 0.001). For deltamethrin, knockdown decreased from 79.7% in the control to 65.0% at 0.001 mg a.i./L (OR = 0.47, 95% CI: 0.33–0.68, *p* < 0.001) and 66.7% at 0.0003 mg a.i./L (OR = 0.51, 95% CI: 0.35–0.74, *p* < 0.001).

In the second generation, knockdown results were similar between PPF-exposed and control groups for both insecticides. For permethrin, KDT_60_ was 27.3% in the control, compared with 30.0% at 0.001 mg a.i./L (*p* = 0.810) and 27.7% at 0.0003 mg a.i./L (*p* = 0.952). For deltamethrin, knockdown was 77.3% in the control and remained comparable at 74.0% (*p* = 0.342) and 75.3% (*p* = 0.565).

In the third generation, responses again showed no significant differences. Permethrin knockdown was 27.3% in the control, similar to 29.0% at 0.001 mg a.i./L (*p* = 0.904) and 31.0% at 0.0003 mg a.i./L (*p* = 0.629). For deltamethrin, control knockdown was 78.7%, with comparable values at 75.7% (*p* = 0.557) and 77.7% (*p* = 0.767) (Table 2).

### 3.3. Effect of Sublethal Dose of Pyriproxyfen on Body Size, Fecundity, and Fertility

**Figure 2:**
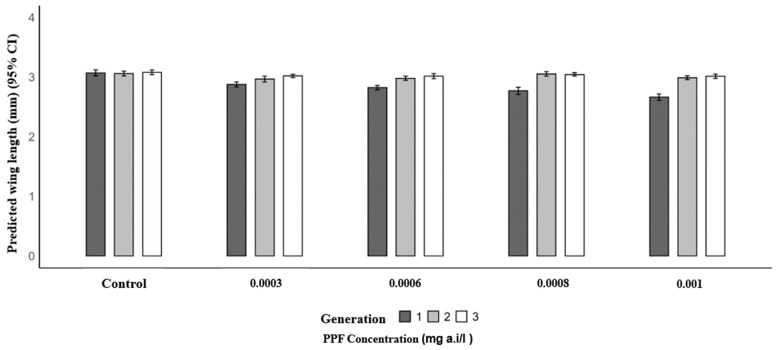
Predicted wing length (mm) of *Anopheles arabiensis* mosquitoes reared across three successive generations under different concentrations of PPF (mg of active ingredient per liter, i.e., mg a.i./L) and a control group.

Mosquitoes from PPF-exposed larvae in the first generation had significantly shorter mean wing lengths compared to the control (3.07 mm). The mean wing lengths were 2.88 mm at 0.0003 mg a.i./L (*p* = 0.004), 2.82 mm at 0.0006 mg a.i./L (*p* < 0.001), 2.77 mm at 0.0008 mg a.i./L (*p* < 0.001), and 2.66 mm at 0.001 mg a.i./L (*p* < 0.001). In the second generation, wing lengths were similar to the control (3.06 mm), with values of 3.05 mm at 0.0003 mg a.i./L (*p* = 0.098), 2.98 mm at 0.0006 mg a.i./L (*p* = 0.157), 3.05 mm at 0.0008 mg a.i./L (*p* = 0.907), and 2.99 mm at 0.001 mg a.i./L (*p* = 0.216). In the third generation, no significant differences were observed, with mean wing lengths of 3.02 mm (0.0003 mg a.i./L, *p* = 0.235), 3.02 mm (0.0006 mg a.i./L, *p* = 0.210), 3.04 mm (0.0008 mg a.i./L, *p* = 0.470), and 3.01 mm (0.001 mg a.i./L, *p* = 0.187) compared to the control (3.08 mm) (Table 3 and Figure 2).

**Figure 3:**
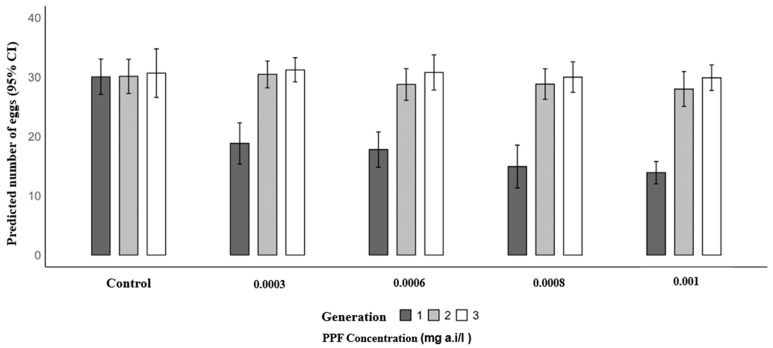
Predicted number of eggs laid of *Anopheles arabiensis* mosquitoes reared across three successive generations under different concentrations of PPF (mg of active ingredient per liter, i.e., mg a.i./L) and a control group.

A similar trend was observed for mean eggs laid. In the first generation, the mean number of eggs per female significantly decreased in PPF-exposed groups compared to the control (30.07): 18.83 at 0.0003 mg a.i./L (RR = 0.63; 95% CI: 0.56–0.70; *p* < 0.001), 17.80 at 0.0006 mg a.i./L (RR = 0.59; 95% CI: 0.53–0.66; *p* < 0.001), 14.93 at 0.0008 mg a.i./L (RR = 0.50; 95% CI: 0.44–0.56; *p* < 0.001), and 13.90 at 0.001 mg a.i./L (RR = 0.46; 95% CI: 0.41–0.52; *p* < 0.001). In the second generation, mean egg production was similar to the control (30.13), with 30.47 at 0.0003 mg a.i./L (*p* = 0.815), 28.77 at 0.0006 mg a.i./L (*p* = 0.329), 28.83 at 0.0008 mg a.i./L (*p* = 0.354), and 28.00 at 0.001 mg a.i./L (*p* = 0.125). In the third generation, no significant differences were observed, with control values of 30.67 and mean egg production of 31.23 at 0.0003 mg a.i./L (*p* = 0.693), 30.80 at 0.0006 mg a.i./L (*p* = 0.926), 30.00 at 0.0008 mg a.i./L (*p* = 0.639), and 29.90 at 0.001 mg a.i./L (*p* = 0.590) (Table 4 and Figure 3).

**Figure 4:**
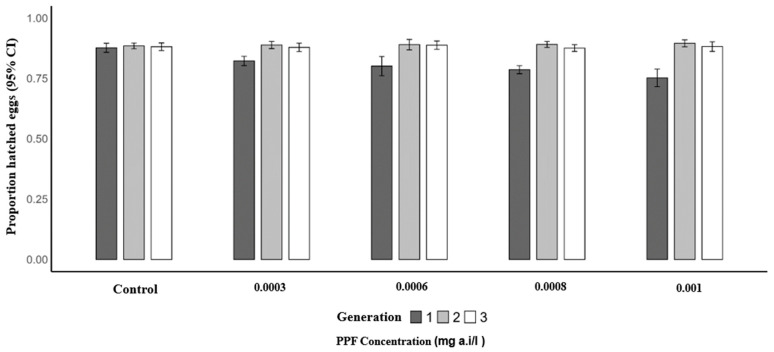
Proportion of hatched eggs of *Anopheles arabiensis* mosquitoes reared across three successive generations under different concentrations of PPF (mg of active ingredient per liter, i.e., mg a.i./L) and a control group.

Similarly, the mean proportion of hatched eggs per female was significantly lower in the first generation of PPF-exposed mosquitoes compared to the control (0.87). The predicted mean proportions were 0.82 at 0.0003 mg a.i./L (OR = 0.66; 95% CI: 0.49–0.88; *p* = 0.005), 0.82 at 0.0006 mg a.i./L (OR = 0.64; 95% CI: 0.48–0.86; *p* = 0.003), 0.79 at 0.0008 mg a.i./L (OR = 0.54; 95% CI: 0.40–0.73; *p* = 0.001), and 0.79 at 0.001 mg a.i./L (OR = 0.55; 95% CI: 0.40–0.75; *p* = 0.001). In the second generation, mean proportions remained similar to the control (0.88), with 0.89 at 0.0003 mg a.i./L (*p* = 0.932), 0.89 at 0.0006 mg a.i./L (*p* = 0.925), 0.89 at 0.0008 mg a.i./L (*p* = 0.565), and 0.88 at 0.001 mg a.i./L (*p* = 0.358). In the third generation, no significant differences were observed, with control proportions of 0.88 and mean proportions of 0.87 at 0.0003 mg a.i./L (*p* = 0.574), 0.89 at 0.0006 mg a.i./L (*p* = 0.690), 0.88 at 0.0008 mg a.i./L (*p* = 0.807), and 0.88 at 0.001 mg a.i./L (*p* = 0.677) (Table 5 and Figure 4).

## 4. Discussion

Identifying new insecticides and innovative delivery methods is a public health priority due to the increasing spread of pyrethroid resistance among disease transmitting mosquitoes [11,19,20,35]. Pyriproxyfen, which is a juvenile hormone analog, is one such insecticide known to induce sterilization and emergence inhibition in exposed mosquitoes [35,36,37]. Previous studies have examined the combined effects of PPF and pyrethroids to understand its interactions with conventional vector control tools like LLINs and IRS [22,23,24,25,26,27,28]. In this context, the present study investigated the effects of sublethal PPF exposure on *An. arabiensis* pyrethroid susceptibility across three generations, and the effect of such exposure on mosquito fecundity, fertility, and body size. We examined these interactions in situations where the emerged adults were exposed to the pyrethroid insecticide class (permethrin and deltamethrin) that is commonly used for adult mosquito control.

The findings show that PPF exposure does not lead to heritable insecticide resistance, but it induces a temporary increase in tolerance in the first generation. However, in the absence of continuous PPF exposure, the second and third generations exhibited susceptibility, fecundity, fertility, and body size comparable to the control group. These results indicate that a single larval exposure to PPF produces transient effects that are not heritable, and that assessing three generations is sufficient to capture its transgenerational impact. As hypothesized, exposure to 3.75% permethrin resulted in 100% knockdown and mortality across all generations, regardless of PPF exposure. These results align with those of Opiyo et al., who reported increased resistance in *An. arabiensis* following sublethal PPF exposure [23]. This study suggests that pyrethroid resistance associated with aquatic exposure to PPF may be restricted to the first generation and is not carried over to subsequent mosquito generations. The observed response likely reflects temporary tolerance rather than true resistance, which arises from the selection of heritable traits within mosquito populations. Cross-resistance between PPF and other insecticide classes against methoxyfenozide (diacylhydrazines), such as cyromazine (triazines), and lufenuron (benzoylureas) [38], has been reported in housefly *Musca domestica*. Resistance to PPF in the greenhouse whitefly *Trialeurodes vaporariorum* has been linked to overexpression of the cytochrome P450 gene CYP4G61, highlighting the potential role of metabolic mechanisms [26].

Some studies suggest that interactions between PPF and pyrethroids could upregulate detoxification enzymes, such as cytochrome P450s, which may metabolize both insecticide classes in *An. arabiensis* [22,23], raising concerns about cross-resistance. However, this effect might be dose-dependent with continuous exposure of mosquitoes to PPF. Prolonged contact with sublethal concentrations of insecticides and domestic pollutants, such as hydrogen peroxide and soap detergent, act as selective pressure that favors resistance traits. For instance, Shayo et al. reported increased tolerance in both susceptible *An. gambiae* and pyrethroid-resistant *An. arabiensis* following exposure to domestic pollutants such as hydrogen peroxide and soap detergent [39]. Similarly, Shilla et al. found enhanced insecticide tolerance in susceptible *An. gambiae* after exposure to microplastics combined with insecticides [40]. These findings highlight the importance of considering environmental co-factors when assessing resistance development.

Because mosquitoes contaminated with PPF through autodissemination become temporarily more tolerant to pyrethroids, this could reduce the short-term efficacy of pyrethroid-based interventions. A key strategy to address this is the rotation of insecticides with different modes of action, which helps maintain overall vector control effectiveness while minimizing potential negative interactions with PPF-based tools. When integrated into a broader management program, PPF interventions provide additional value by targeting immature mosquito stages and complementing existing control measures. Notably, when permethrin was tested at a high dose of 3.75%, all mosquitoes, whether pre-exposed to PPF or not, were fully susceptible across all three generations. This confirms that high-dose intensity assays are effective in resistant populations.

Nevertheless, exposure to sublethal doses of PPF during the larval stage significantly affected key reproductive and fitness parameters in the first generation of *An. arabiensis* mosquitoes. Fecundity and fertility were both markedly reduced, with egg-laying and hatching rates significantly lower in PPF-exposed groups compared to controls. Additionally, exposed mosquitoes had shorter wing lengths, indicating reduced body size, which is a known proxy for overall fitness and survival. These effects were not observed in the second and third generations, suggesting that the impact of PPF is transient and confined to direct exposure. Our findings are consistent with previous studies demonstrating PPF’s ability to impair mosquito reproduction and development [35,41,42,43,44,45,46,47]. The reduction on fecundity and fertility could be due to the fact that PFF mimics the action of JH at a time that it should not be present, affecting the vitellogenesis or some other step in the formation of eggs [35,37,42,43]. Similar effects have been reported in LLINs co-treated with PPF, where resistant mosquitoes were sterilized upon exposure to Olyset Duo [28,48,49]. These findings highlight the added value of PPF in vector control, as it can induce sterility even in resistant mosquitoes. The sterilizing effect of PPF either at larval or adult exposure supports its potential as a complementary tool for managing resistant mosquito populations and interrupting transmission.

The smaller body sizes observed in the exposed groups are likely a consequence of PPF induced stress during larval development, although other factors such as chemical exposure or larval competition are also known to produce smaller adults [44,46,50]. From a vector control perspective, PPF exposure is particularly valuable, as it can reduce mosquito population growth and vectorial capacity even without causing immediate mortality. By limiting reproductive output and reducing adult fitness, PPF contributes to an integrated approach that suppresses vector populations and complements existing interventions.

The limitations of this study include: (1) Only the first generation of mosquitoes was exposed to PPF, while subsequent generations were reared without further exposure. Future studies should extend across multiple generations with repeated PPF exposure to better capture long-term effects. (2) Further investigations are needed to assess delayed effects on mosquitoes beyond the 24 h period following PPF pre-exposure. Additionally, it remains unclear whether similar sublethal effects, particularly the temporary increase in pyrethroid tolerance, would occur in *Anopheles gambiae s.s.* or *Anopheles funestus*. These species differ in larval ecology, behavior, and resistance mechanisms, which could influence their responses to PPF, emphasizing the need for species-specific assessments in future studies. (3) It should be noted that this study assessed phenotypic resistance, which reflects the observable response of mosquitoes to insecticide exposure. While the results suggest that metabolic resistance may play a role, it is likely that other underlying mechanisms also contribute to the observed tolerance. Therefore, further investigations, including molecular and gene expression analyses, are necessary to identify the specific genes and pathways responsible for the upregulation of detoxification enzymes and to clarify the mechanisms driving resistance in these mosquito populations. Moreover, studies should be conducted to examine whether field applications of PPF, either by larviciding or other means, exacerbate pyrethroid resistance in areas where such resistance already exists in wild vector populations. This study provides important insights into the transient nature of PPF-induced effects on insecticide susceptibility and mosquito fitness, supporting its potential role as a complementary tool in integrated vector management strategies.

## 5. Conclusions

This study demonstrates that sublethal exposure of *Anopheles arabiensis* larvae to PPF temporarily reduces adult susceptibility to pyrethroids and negatively affects key fitness traits in the first generation, including wing size, egg production, and hatching success. Importantly, these effects were not observed in subsequent generations, indicating that the impact of PPF is transient. These findings support the strategic use of PPF in integrated vector management, suggesting that it can be rotated with pyrethroids to help manage resistance while maintaining the effectiveness of existing control interventions.

## Figures and Tables

**Table 1:** Mortality of resistant *Anopheles arabiensis* emerging from varying concentrations of pyriproxyfen and exposed to permethrin and deltamethrin.

Insecticides	Generations	PPF Conc. (mg a.i/L)	OR [95% CI]	Mean Mortality (%) ± SD	*p* Value	Susceptibility Status
Permethrin	1	No PPF	1	22.67 ± 3.11	–	R
		0.001	0.66 (0.52–0.82)	3 ± 2.49	*p* < 0.001	R
		0.0003	0.77 (0.60–0.97)	11 ± 4.86	0.026	R
	2	No PPF	1	22.67 ± 3.94	–	R
		0.001	0.99 (0.79–1.25)	23 ± 3.86	0.953	R
		0.0003	0.97 (0.77–1.23)	22 ± 3.19	0.813	R
	3	No PPF	1	25 ± 4.22	–	R
		0.001	1.05 (0.83–1.33)	24.67 ± 4.77	0.694	R
		0.0003	1.01 (0.80–1.27)	22.67 ± 4.29	0.953	R
Deltamethrin	1	No PPF	1	62.67 ± 5.99	–	R
		0.001	0.18 (0.13–0.26)	23.33 ± 7.78	*p* < 0.001	R
		0.0003	0.34 (0.24–0.47)	36 ± 11.05	*p* < 0.001	R
	2	No PPF	1	60.33 ± 7.33	–	R
		0.001	0.99 (0.71–1.37)	60 ± 14.77	0.933	R
		0.0003	0.92 (0.66–1.28)	58.33 ± 3.98	0.617	R
	3	No PPF	1	62 ± 6.27	–	R
		0.001	0.89 (0.64–1.24)	61.33 ± 6.23	0.867	R
		0.0003	0.97 (0.70–1.35)	59.33 ± 9.16	0.504	R

Key: SD = Standard deviation; OR = Odds ratio; CI = Confidence Interval.

**Table 2:** Knockdown at 60 min in resistant *Anopheles arabiensis* emerging from varying concentrations of pyriproxyfen and exposed to permethrin and deltamethrin.

Insecticides	Generations	PPF Conc. (mg a.i/L)	KDT_60_ (min) (%) ± SD	OR [95% CI]	*p* Value
Permethrin	1	No PPF	41.3 ± 7.9	1	–
		0.001	3.3 ± 2.3	0.44 (0.35–0.56)	*p* < 0.001
		0.0003	9.3 ± 4.3	0.50 (0.39–0.64)	*p* < 0.001
	2	No PPF	27.3 ± 6.8	1	–
		0.001	30 ± 7.1	1.03 (0.81–1.30)	0.810
		0.0003	27.7 ± 8.6	1.01 (0.80–1.27)	0.952
	3	No PPF	27.3 ± 8.3	1	–
		0.001	29 ± 6.8	1.01 (0.80–1.29)	0.904
		0.0003	31 ± 4.6.	1.06 (0.84–1.34)	0.629
Deltamethrin	1	No PPF	79.7 ± 6.9	1	–
		0.001	65 ± 3.5	0.47 (0.33–0.68)	*p* < 0.001
		0.0003	66.7 ± 4.9	0.51 (0.35–0.74)	*p* < 0.001
	2	No PPF	77.3± 6.5	1	–
		0.001	74 ± 4	0.83 (0.57–1.61)	0.342
		0.0003	75.3 ± 4.1	0.90 (0.61–1.30)	0.565
	3	No PPF	78.7 ± 6.2	1	–
		0.001	75.7 ± 3.3	0.89 (0.61–1.31)	0.557
		0.0003	77.7 ± 7.3	0.94 (0.64–1.39)	0.767

Key: SD = Standard deviation; OR = Odds ratio; CI = Confidence Interval.

**Table 3:** Predicted mean wing length of *Anopheles arabiensis* between control and pyriproxyfen exposed groups across varying concentrations.

Generations	PPF Concentrations	Predicted Mean [95% CI]	Estimate [95% CI]	*p* Value
	(mg a.i/L)	(mm) *		
1	Control	3.07 (2.99–3.15)	1	–
	0.0003	2.88 (2.88–2.80)	0.94 (0.90–0.98)	0.004
	0.0006	2.82 (2.74–2.90)	0.92 (0.88–0.96)	*p* < 0.001
	0.0008	2.77 (2.69–2.85)	0.90 (0.86–0.95)	*p* < 0.001
	0.001	2.66 (2.58–2.74)	0.87 (0.87–0.91)	*p* < 0.001
2	Control	3.06 (2.98–3.14)	1	–
	0.0003	3.05 (2.97–3.13)	0.97 (0.93–1.01)	0.098
	0.0006	2.98 (2.90–3.06)	0.97 (0.94–1.01)	0.157
	0.0008	3.05 (2.97–3.13)	1.00 (0.96–1.04)	0.9 07
	0.001	2.99 (2.91–3.07)	0.98 (0.94–1.01)	0.216
3	Control	3.08 (3.00–3.16)	1	–
	0.0003	3.02 (2.94–3.10)	0.98 (0.95–0.01)	0.235
	0.0006	3.02 (2.94–3.10	0.98 (0.95–0.01)	0.210
	0.0008	3.04 (2.96–3.12)	0.99 (0.96–0.02)	0.470
	0.001	3.01 (2.93–3.09)	0.98 (0.95–0.01)	0.187

Key: CI; Confidence Interval * predicted values were obtained from a GLMs model.

**Table 4:** Predicted mean eggs laid of *Anopheles arabiensis* between control and pyriproxyfen exposed groups across varying concentrations.

Generations	PPF Concentrations.	Predicted Mean [95% CI] *	RR [95% CI]	*p* Value
	(mg a.i/L)			
1	Control	30.07 (28.17–32.09)	1	–
	0.0003	18.83 (17.34–20.45)	0.63 (0.56–0.70)	*p* < 0.001
	0.0006	17.80 (16.35–19.38)	0.59 (0.53–0.66)	*p* < 0.001
	0.0008	14.93 (13.61–16.38)	0.50 (0.44–0.56)	*p* < 0.001
	0.001	13.90 (12.63–15.30)	0.46 (0.41– 0.52)	*p* < 0.001
2	Control	30.13 (28.23–32.16)	1	–
	0.0003	30.47 (28.55–32.51)	1.01 (0.92–1.11)	0.815
	0.0006	28.77 (26.91–30.75)	0.95 (0.87–1.05)	0.329
	0.0008	28.83 (26.97–30.82)	0.96 (0.87–1.05)	0.354
	0.001	28.00 (26.17–29.96)	0.93 (0.85–1.02)	0.125
3	Control	30.67 (28.75–32.71)	1	–
	0.0003	31.23 (29.30–33.30)	1.02 (0.93–1.12)	0.693
	0.0006	30.80 (28.88–32.85)	1.00 (0.92–1.10)	0.926
	0.0008	30.00 (28.10–32.03)	0.98 (0.89–1.07)	0.639
	0.001	29.90 (28.01–31.92)	0.97 (0.89–1.07)	0.590

Key: CI; Confidence Interval, RR; Relative Risk * predicted values were obtained from a GLMs model.

**Table 5:** Predicted mean hatching rate of *Anopheles arabiensis* eggs between control and pyriproxyfen exposed groups across varying concentrations.

Generations	PPF Concentrations.	Predicted Mean Proportion [95% CI] *	OR [95% CI]	*p* Value
	(mg a.i/L)			
1	Control	0.87 (0.85–0.89)	1	–
	0.0003	0.82 (0.79–0.85)	0.66 (0.49–0.88)	0.005
	0.0006	0.82 (0.78–0.85)	0.64 (0.48–0.86)	0.003
	0.0008	0.79 (0.75–0.82)	0.54 (0.40–0.73)	0.001
	0.001	0.79 (0.75–0.83)	0.55 (0.40–0.75)	0.001
2	Control	0.88 (0.86–0.90)	1	–
	0.0003	0.89 (0.86–0.89)	1.01 (0.76–1.35)	0.932
	0.0006	0.89 (0.86–0.89)	1.01 (0.76–1.36)	0.925
	0.0008	0.89 (0.87–0.91)	1.09 (0.81–1.47)	0.565
	0.001	0.88 (0.85–0.90)	1.15 (0.55–1.86)	0.358
3	Control	0.88 (0.86–0.90)	1	–
	0.0003	0.87 (0.85–0.89)	0.92 (0.97–1.22)	0.574
	0.0006	0.89 (0.87–0.91)	1.06 (0.80–1.41)	0.690
	0.0008	0.88 (0.86–0.90)	0.97 (073–1.28)	0.807
	0.001	0.88 (0.85–0.90)	0.94 (0.71–1.25)	0.677

Key: CI; Confidence Interval, RR; Relative Risk * predicted values were obtained from a binomial GLMs model.

## Data Availability

The data presented in this study are available on request from the corresponding authors due to institutional data privacy.

## Abbreviations

a.i	Active ingredient
DHS-MIS	Demographic Health Survey Malaria Indicator Survey
GTS	Global Technical Strategy
IGR	Insecticide Growth Regulator
IVM	Integrated Vector Management
IRS	Indoor Residual Spray
LC	Lethal Concentration
LLINs	Long Lasting Insecticide Treated nets
LSM	Larval Source Management
NMSP	National Malaria Strategic Plan
PPF	Pyriproxyfen
SFS	Semi Field System
WHO	World Health Organization
